# The Present Position of Radium Therapy
*A Paper read at a Meeting of the Bristol Medico-Chirurgical Society, held in the University of Bristol on 10th May, 1933.


**Published:** 1933

**Authors:** Sylvia B. Wigoder

**Affiliations:** Radium Officer, National Radium Centre, Bristol


### THE PRESENT POSITION OF RADIUM THERAPY.*

BY

Sylvia B. Wigoder, M.D., Ph.D., D.M.R.E.,
*Radium Officer, National Radium Centre, Bristol.*

---

Radium has been described (I have forgotten by whom,) as the most potent element which has been yet discovered for the production of discontent in the medical profession. It was hailed at first as a panacea for all ills. Although it has not fulfilled the hopes based upon it by those early enthusiasts, it has opened up new fields in the treatment of disease, especially malignant disease, but even there, despite improved technique, I do not think that radium is at present a substitute for surgery. The radium needle should be looked upon, not as a competitor to the knife, but as a partner and an added weapon in the fight against cancer.

Radium therapy should be restricted to inoperable cases or those cases where operation is attended by great risk, such as Wertheim's hysterectomy, or associated with mutilation, such as removal of the tongue. Of the inoperable cases those that are very far advanced with widespread metastasis, bony

* A Paper read at a Meeting of the Bristol Medico-Chirurgical Society, held in the University of Bristol on 10th May, 1933.

T

involvement, much sepsis and marked wasting are generally unsuitable; while patients suffering from diabetes, nephritis, syphilis or tuberculosis do not, as a rule, respond well to radium treatment. In addition, accessibility of the lesion, its proximity to vital structures and its histological type modify the results. Adeno-carcinomata are considered radio-resistant and well-differentiated squamous-celled carcinoma less resistant, while non-differentiated epithelioma and the basal-celled carcinoma or rodent ulcer are radio-sensitive. Many of the sarcomata, especially lympho-sarcoma, are extremely radio-sensitive, but unfortunately these growths tend to recur either locally or elsewhere very soon after irradiation.

Roughly about 50 per cent. of the malignant cases treated by radium are locally well, mostly symptom-free at the end of one year. This is a considerable achievement, as the majority of these cases are hopeless from a surgical point of view, and so, were it not for radium treatment, most of them would have been dead and certainly none of them would have been symptom-free at the end of a year. There is, as William Mayo says, a great difficulty in evaluating radium treatment, since it is used so largely in treating inoperable cancer or cancer following operation, where the most that can be expected is palliation. The thoughtless, therefore, give radium little credit for what it actually accomplishes in these cases and, disappointed that the impossible has not been accomplished, minimize its value. I mention one year as the period of freedom from symptoms, not because I believe that recurrences either occur within one year or not at all, but because the patient and his doctor are more interested in the immediate effects of radium treatment and are less concerned with what will happen five years hence; and secondly, techniques have altered so rapidly within the last few years that reliable five-year statistics are difficult to obtain.

At the outset I should like to make it clear that the results mentioned in this paper are the average results obtained from a number of centres. Gordon-Watson, I know, has obtained good results in carcinoma of the rectum, and Geoffrey Keynes's results in carcinoma of the breast are excellent; but these are exceptional, and at the majority of centres results are less promising when these regions are treated.

In spite of advances in the knowledge of the biological and physical properties of radium, the conditions governing the vulnerability of normal and malignant cells are still, to a large extent, problematical, and we still do not know why two apparently similar cancers with apparently similar histological structure will react in a dissimilar manner to radium. The histologist and cytologist have worked long and hard at the problem, and the solution, I think, now lies in the hands of the bio-chemist and bio-physicist. However, just as the majority of practitioners do not consciously think of the exact *modus operandi* of the drugs they use, we radiologists use radium even if the processes initiated in the tissues by radiation are far from being understood.

There are four main radium techniques. The *first* is that of interstitial needling, in which radium is inserted surgically into the diseased tissues. This method is that most often employed at present, but requires a general anæsthetic; the lesion is surrounded by needles which are left in place for six to fourteen days, depending on the histological type of lesion, the strength and filtration of the needles. *The second method,* the intracavitary method, is that in which radium is placed in a natural cavity of the body, for example, in the body of the uterus or in the œsophagus. This treatment rarely lasts for longer than a week. *The third method,* that of surface treatment, consists of covering the surface of the lesion with radium, which may be directly applied to it or separated from it by $\frac{1}{4}$ to 4 cm. of some material such as wax or rubber. Treatment here may last from a few hours up till ten to fourteen days, depending not only on the type of lesion and the concentration of the radium, but also on the distance of the lesion from the radium. A small superficial lesion will, of course, have the radium closely applied to it. *The fourth method* of treatment is the much discussed distance radium or mass irradiation therapy, when a bomb containing a large quantity of radium is placed at some inches from the body. There are many conscientious enthusiasts who advocate the use of the bomb: equally many decry it. Results on the Continent have, on the whole, been good. The lack of success in England may, perhaps, have been due to our lack of proficiency with smaller units, but it seems to me that before we can use huge quantities, such as 15 grammes, we will want not only larger hospitals to house the radium, but bigger and stronger patients to stand the treatment. There is no definite therapeutic difference between the use of element radium and its emanation radon, except that radon, being a gas, can be collected, compressed and sealed in small containers known as seeds.

Shortly after the beginning of radium treatment the lesion becomes covered with a smooth, greenish-yellow fibrinous exudate not unlike a diphtheritic membrane, which increases for about three weeks, when it gradually fades away from the edge, being replaced by healthy tissue. When radium is applied to the unbroken skin, *e.g.* in the treatment of a deep-seated mass, this reaction is preceded by reddening of the skin and desquamation. This typical reaction is not a burn, although when it appears on what was formerly unbroken skin the uninitiated may refer to it as a burn. Sometimes, however, the reaction may last longer, and in the case of over-dosage may persist for months with the formation of an ulcer. The radio-necrotic ulcer or burn has usually a sharp, punched-out edge instead of the smooth edge of the normal reaction. It occurs at the centre of the site treated, may be deep, and is often very painful. The best treatment is removal by diathermy. The presence of a radio-necrosis is not always due to bad technique, any trauma, intense heat or cold, etc., may cause it, and it may occasionally be due to a personal idiosyncrasy on the part of the patient.

Lesions of the *Skin* may be treated by either surface or interstitial radium. Results on the whole are equally good. These lesions are usually radio-sensitive, and, provided bone is not involved, good and lasting results may be expected in the majority of cases. Radium therapy here is definitely superior to surgery, since there is no mutilation, the lesion being replaced by a supple scar. Should glandular metastasis arise in association with a lesion in any part of the body it should be dealt with surgically. Epitheliomata on old lupus do not give such good results, as a great number of them recur after a year. Sometimes it is worth while treating this recurrence, but as the skin is very devitalized, another occurs rapidly, or necrosis appears. In rodent ulcer the results are excellent and superior to any other form of treatment.

Lesions of the *Lip* may also be treated by either surface or interstitial treatment. The results here are very good, and up to three-quarters of even the most advanced cases may be alive at the end of the year.

The opinion of most gynæcologists is that radium is preferable to surgery in carcinoma of the *Cervix Uteri,* since there is very little risk and even the most advanced cases derive benefit. As a rule bleeding and discharge become appreciably less and frequently cease, and about half of the cases treated are alive at the end of the year. Sometimes an apparently cured case complains of backache. This may be due to involvement of the ureters in fibrous tissue, a condition which the gynæcologist can sometimes relieve. Treatment usually consists of intra-uterine and vaginal applications for about seven days, either continuously or in separate applications spread out over three to six weeks. Sometimes interstitial needling is used. Post-radium X-ray therapy has increased the number of good results. Unfortunately, results of radium treatment are disappointing in this region if the patient is under forty, and if the case is operable it is advisable to operate. Opinions differ on the value of radium therapy in carcinoma of the *Body of the Uterus.* Some centres report results equal to those obtained by surgery, but I think the majority of centres regard adeno-carcinoma of the body of the uterus as being radio-resistant. In carcinoma of the *Vulva* radium therapy gives bad results. Temporary disappearance of the growth is frequently obtained, but, possibly due to interference with the blood supply, the dose sufficient to kill the growth usually causes a necrosis of the tissues with the formation of a deep, sloughing, painful ulcer.

In carcinoma of the *Mouth, Tongue and Tonsil* results are encouraging, as, with a negligible primary mortality, about half of the cases are alive at the end of the year. If, however, there is bony involvement radium treatment may result in a slow and painful necrosis. When the tongue is absolutely fixed, palliation and some relief from pain is sometimes obtained by applying very large doses of radium for a short length of time. In extremely advanced cases radium is, I think, contra-indicated, as a certain amount of œdema and pain follow treatment, and so, for no useful purpose, the patient's discomfort is aggravated. The majority of lesions on the inside of the *Cheek* seem to arise from an old leukoplakia, and so the results here are disappointing, and less than 50 per cent. are said to be symptom free at the end of one year. Treatment usually consists of interstitial needling, but occasionally surface applicators are used. The latter are said to give better results.

Carcinoma of the *Anus,* being a squamous-celled carcinoma, responds well to radium, and even in very advanced cases treatment may produce temporary and local relief. Carcinoma of the *Rectum*, however, being an adeno-carcinoma, gives on the whole bad results, although occasionally after radium an inoperable case becomes operable. In this site radium may be inserted interstitially into the growth or a central radium applicator may be passed into the rectum, but the latter method is not frequently used nowadays.

The radium treatment of carcinoma of the *Bladder* is still more or less in an experimental stage, but some good results have been reported from the New York Memorial Hospital. Because of their small size radon seeds are more suitable than needles. The usual method of treatment consists of opening the bladder and inserting the radium into the base of the growth, where it remains for about eight days, or, in the case of seeds, for ever.

The value of radium treatment of carcinoma of the *Prostate* is still on trial, and no figures are available from any of the larger centres, although quite good results have been reported by individual surgeons. Needles are usually inserted interstitially through the perineum or bladder.

In the treatment of carcinoma of the *Breast* radium has not realized our earlier hopes, and operation is still the method of choice, perhaps combined with radium. In inoperable cases, or those showing one or two bony nodules, radium (either used in the form of a surface application or interstitially) sometimes produces temporary palliation with disappearance of the mass, but very advanced cases with marked skin or glandular involvement derive no benefit.

Intrinsic carcinoma of the *Larynx* gives very good results when combined with fenestration operation, and palliation has been obtained in some extrinsic carcinomata.

The results from radium treatment of carcinoma of the *Antrum* are gradually improving, due perhaps to a closer co-operation between the radiologist and the surgeon. Treatment consists, as a rule, of opening the antrum and the distribution of needles or seeds around the growth. This may be followed by a surface application on the outside of the face.

Carcinomata of the *Internal Organs* and of the *Alimentary Tract* respond badly to radium. In carcinoma of the œsophagus disappearance of the growth has sometimes occurred, but it recurs in about six months, and similar benefit may be obtained by the insertion of a Souttar's tube without the use of radium.

It is not only in malignant disease that radium has proved of benefit. Indeed, if it had no other field of usefulness except its ability to control uterine hæmorrhage, it would be a remarkable therapeutic agent on that basis alone. But, in addition to these uses, it has proved of benefit in many skin lesions such as tuberculous ulcers, warts and corns, and is at present being tried out, with some measure of success, in other conditions, such as painful bursas, goitres, etc.

In writing this paper I have kept in front of me the advice of Pasteur: "Your enthusiasm, which is the fruit of your earlier years, keep it, but let it be attended by a rigorous self-control"; and I have tried to show you the real worth of radium by being neither unduly optimistic nor unduly pessimistic.

Whatever may be the ultimate place that radium will gain and hold in the armoury of the surgeon and the radiologist, it has already certainly proved to be a most useful palliative agent and has a definite curative value in a good many cases.

